# Clinical Events Associated with Acupuncture Intervention for the Treatment of Chronic Inflammation Associated Disorders

**DOI:** 10.1155/2020/2675785

**Published:** 2020-06-27

**Authors:** Hua Bai, Senlei Xu, Qiulan Wu, Shanshan Xu, Ke Sun, Jiahong Wu, Xuefeng Xia, Yuchen Liu, Hongru Zhang, Shengfeng Lu

**Affiliations:** ^1^The Second Clinical Medical College, Nanjing University of Chinese Medicine, Nanjing, 210023 Jiangsu, China; ^2^Affiliated Hospital of Integrated Traditional Chinese and Western Medicine, Nanjing University of Chinese Medicine, Nanjing, 210028 Jiangsu, China; ^3^Department of Acupuncture, Yuhua Community Health Service Center, Nanjing, 210012 Jiangsu, China

## Abstract

Acupuncture is a key component of Chinese medicine. It describes a series of procedures involving the stimulation of skin through penetration of fine, single-use, sterile needles that result in the release of neurotransmitters. Although its use is on the growing trend, considerable controversy surrounds its value as a therapy. Standard randomized controlled trials that adhere to the accepted criteria should be conducted in the future to ensure the effectiveness of acupuncture. This article summarizes the current evidence regarding the use of acupuncture. It includes a description of the history, mode of operation, treatment of a variety of chronic disorders related to inflammation, and future directions for acupuncture use. Published clinical trials support the view that acupuncture is a possible candidate for the treatment of several chronic inflammation-related disorders.

## 1. Introduction

The anti-inflammatory effect of acupuncture is a key analgesic mechanism of reducing pain [1]. Adverse effects associated with acupuncture are rare and minor [2]. Hence, it is a relatively safe complementary medicine to use when handled by qualified practitioners [3]. Although acupuncture in TCM has been used for the treatment of many disorders, in this review, we took a comprehensive literature survey to determine the clinical trials for its effectiveness in chronic-inflammation associated diseases.

## 2. History of Acupuncture Use

Although acupuncture has been dated back to 3000 years in China, stones and bones aging to 6000 BC have been identified with traces of acupuncture use [4]. Ranging from 1500 to 1700 AD, the Dutch East India Company initiated the concept of acupuncture into European medical practice. The first-ever clinical report on acupuncture was published as “On Acupuncturation.” in the year 1802 by the British physician William Coley. A series of then acupuncture related publications led to positive opinions about acupuncture among the medical community in Europe. The early 1800s saw the entry of acupuncture related publications into US medical journals. After a sequence of fluctuations, the National Institutes of Health (NIH), in 1997, acknowledged acupuncture for its benefits in the field of clinical medicine and recommended it to be included in the scheme of medical schools [5]. In 1979, the World Health Organization (WHO) organized a meeting which identified 43 different diseases that could be treated by acupuncture. It also published a book in 2003 to determine the clinical trials performed related to acupuncture [6].

## 3. Acupuncture-Modus Operandi

A loss of balance between the yin and yang energies is understood as a disease in Chinese medicine. Acupuncture relies on maintaining a balance between yin and yang through the use of several acupoints and 12 meridians. Among the 12 meridians, yin meridians include lung, spleen, heart, kidney, pericardium, and liver. Large intestines, stomach, small intestines, bladder, triple energizer, and gall-bladder are defined as yang meridians. The circulation of *Qi*, a vital life force that flows through the body is supposedly regulated by acupuncture via 12 meridians in a discrete order as mentioned in Chinese medicine body clock [7]. Acupuncture stimulation through the insertion of fine, single-use, sterile needles can result in the release of neurotransmitters such as endorphins, serotonin, enkephalins, gammaaminobutyric acid, norepinephrine, and dopamine [8]. Appropriate use of combinations in acupuncture can result in proper flow of energy through the meridians [9]. The effects of acupuncture are comparable to the effects observed after the use of capsaicin, a bioactive component of chili pepper [10].

## 4. Therapeutic Potential of Acupuncture in Chronic Inflammation Related Disorders

Acupuncture is effective as first-line or adjunct medication for treatment of chronic inflammation associated disorders such as allergic rhinitis, anxiety, asthma, atherosclerosis, and associated myocardial infarction, cancer, rheumatoid arthritis, stress, and Crohn's disease. The intervention is considered to be systemically effective and safe in the management of diseases associated with various regions of the human body [11–19]. Local-distal combination is widely used for intervention using acupuncture, whereas, local administration is effective for a local symptom. Further research on the comparison of effectiveness is warranted [20]. Local-distal acupoints such as Bladder 40 had been identified for specific disorders. These acupoints were found to be effective for the treatment of inflammation-associated disorders in comparison with local administration [21–25]. Dr. Tan's strategy applies a systemic approach to treat a patient. It is a novel approach to manage or cause significant alleviation of several disorders through the use of 12 points [26, 27].

### 4.1. Allergic Rhinitis

Allergic rhinitis is the most common respiratory disease. Acupuncture is a nasal obstruction and sneezing of 61 persistent allergic rhinitis patients improved at week 2 of acupuncture treatment at the 5th Affiliated Hospital of Sun Yat-Sen University, China. The sphenopalatine acupoint located in the cheek near Xiaguan was used for stimulation. The total nasal symptom score was significantly lower compared to the control [28]. Twenty-six patients aging 18 to 70 were selected to study the effects of acupuncture on seasonal allergic rhinitis at the RMIT University, Victoria, Australia. Ying Xiang, Yintang, and Fengchi were the acupoints used for stimulation. A significant improvement was observed in sneezing, watery rhinorrhoea, nasal congestion, itching nose (symptoms associated with nose), itching eyes, and eye-watering (nonnasal symptoms) [29].

A nonrandomized controlled design was used at Zhejiang Hospital, Hangzhou, China, to evaluate the effect and safety of acupuncture therapy on participants with moderate to severe allergic rhinitis. The thirty-six patients treated with acupuncture recovered effectively in equivalence to the effects of oral cetirizine hydrochloride tablets after four weeks of treatment. The acupoints used were Ying Xiang, Shang Xing, Fengchi, Quchi, Xue Hai, Fei Shu, Ge Shu, and Pishu. The scores for sneezing, runny nose, stuffy nose, nasal itching, and turbinate edema were similar among both the groups [30]. A multicenter, randomized, parallel-controlled study was conducted based at two centers in South Korea and one in China. The acupoints used were bilateral He Gu, Ying Xiang, Sibai, Zu San Li, unilateral Yintang, and Shang Xing. The total nasal and nonnasal symptom score of 97 patients treated with acupuncture was reduced in comparison to control groups after 4 weeks of treatment [31]. All these studies indicate that allergic rhinitis could be treated effectively by using acupuncture.

### 4.2. Asthma

Asthma is considered to be one among the widespread chronic respiratory diseases [32]. This childhood disorder causes limitations in the airflow of the airways in the 300 million affected individuals around the world and kills an estimate 346,000 every year [14]. Acupuncture has a long tradition in the treatment of asthma [33]. In an interesting study conducted at Jinan Hospital of TCM, China, acupuncture at Yuji meridian relieved the acute attack of bronchial asthma in two hundred and eighty-nine cases. The effects of 30-minute needle-retaining were comparable to the effects of 200 *μ*g salbutamol aerosol [34]. Improved pulmonary function and reduced asthma attack were observed in ninety acute stage asthma cases at the Tianjin City Hospital of TCM, China, after acupuncture treatment [35]. Acupuncture significantly improved the symptoms of asthma and the Visual Analogue Scale of dyspnea in six patients with moderate to severe persistent bronchial asthma at the Department of Internal Medicine, Meiji University of Oriental Medicine, Japan. The combination of meridian points used for the treatment was Zhongfu, Chize, Tai Yuan, Guan Yuan, Zhong Wan, Fei Shu, Pishu, and Shen Shu [36]. Zhaohai and Lie Que are opening and coupling points for Ren Mai [37]. These points are used to maintain homeostasis of the lung and kidneys [38]. Although Zhaohai is not widely used for the treatment of asthma, and Lie Que is a connecting point for lung channel which is used widely in the treatment of asthma over the recent decades. It is an essential point in Chinese acupuncture according to the clinical trials for the treatment of asthma [39, 40]. Although several local and distal acupoints including Back Shu are used to tonify and sedate heat and cold symptoms of lung in Sa-Ahm's acupuncture, the clinical use of such methods is still a concern. Yin meridians of water such as kidney meridian should be suppressed for maintaining a systemic balance [41]. Yin and yang meridians such as Shao Shang and Shang Yang are used to clear lung heat [42]. Fei Shu and acupoints of other meridians could be used to treat complications such as COPD [43].

These studies show that acupuncture could be used in the effective treatment of asthma through the stimulation of several meridian points.

### 4.3. Atherosclerosis

Electroacupuncture pretreatment was given at the bilateral Neiguan, Lieque, and Yunmen acupoints for 30 minutes each day for five consecutive days before surgery in sixty patients of the Fourth Military Medical University, Xi'an, China, suffering from heart valve disease. Limited troponin I release, reduction in ICU stay time, and decreased use of inotropic agents were the postoperative effects observed in the treated patients [44]. Thirty-five cases of The First Affiliated Hospital of Tianjin University of TCM, China, were randomized to receive acupuncture and moxibustion at certain acupoints including Renying, Neiguan, and Zu San Li. The increase in size of the common carotid artery, reduction of intima-media thickness, accelerated peak systolic, and end-diastolic velocities, decreased pulsatility index, and resistance index were the effects observed after treatment [45]. 204 patients with coronary artery disease from 5 multicentered hospitals in China were registered for a clinical trial using electroacupuncture. All these patients under age 18 intended to receive percutaneous coronary intervention were treated with electroacupuncture for 30 minutes before the coronary intervention procedure. Stimulation at Neiguan and Ximen acupoints resulted in a decrease of the rate of major adverse cardiac/cerebrovascular events after a two-year follow-up [46].

Neiguan is the most used acupoint for the treatment of cardiac ailments by involving several mechanisms that improve angiogenesis and reduce apoptosis and calcium overload [47]. Zu San Li and auricular acupoints are other commonly used ones [48]. These studies indicate that acupuncture could be used in the improvement of blood flow and to alleviate the symptoms of atherosclerosis, thereby preventing their occurrence and development in vulnerable individuals.

### 4.4. Cancer

A case study was performed at Oregon College of Oriental Medicine, Oregon, USA, with a patient suffering from ductal carcinoma in situ. She received acupuncture treatment by stimulation at the Tai Chong (19 times), followed by Zu San Li, He Gu, Qi Hai, Fenglong, and San Yin Jiao along with the use of four TCM extracts and a prescribed diet. This noninvasive form of breast carcinoma regressed by 75% over a treatment period of 15 months [49]. Zu San Li, Neiguan, Ge Shu, Fei Shu, Guan Yuan, and Shen Shu were the acupoints represented in their order of frequency of use in the treatment of lung cancer. A significant increase of IL-2, CD3+, and CD4+ T cells and NK cells were observed after the stimulation of these acupoints [50]. Electroacupuncture was clinically effective in the treatment of 55.6% of 36 patients with massive liver cancer at the China-Japan Friendship Hospital, Beijing, China [51]. IL-2 level and NK cell activities increased in peripheral blood of 25 patients with malignant tumors after acupuncture stimulation of 30 minutes for 10 days at acupoints Zu San Li, Quchi, and Qi Hai [52]. These studies determine that acupuncture could be effective in the treatment of patients with cancers of various origins.

### 4.5. Rheumatoid Arthritis

Thirty-six rheumatoid arthritis patients (mean age-58 ± 10 years; disease duration-9.3 ± 6.4 years) of the Prince of Wales Hospital were recruited for a randomized, double-blind, placebo-controlled trial at The Institute of Chinese Medicine at The Chinese University of Hong Kong. All the patients received electroacupuncture and acupuncture for 20 sessions of two 40 minute—sessions every week over a 10-week period. Quchi, Waiguan, He Gu, Zu San Li, Yang Ling Quan, and Xuan Zhong were the acupoints used for the study. The pain score reduced in the treatment groups [17]. Laser acupuncture was employed at Tai Chong, Tianshu, Zu San Li, Houxi, Wan Gu, He Gu, Qu Chi, San Yin Jiao, Ying Ling Quan, Jing Men, Yang Ling Quan, and Shen Men acupoints of 15 arthritis patients aged 60-70 at the outpatient clinic of Kasr El Ainy Medical School and National Institute of Laser Enhanced Sciences, Cairo University, Egypt. Interleukin-6 and malondialdehyde levels decreased, whereas ATP levels and pain-free range of motion at joints increased [53]. Laser acupuncture (3 days/week for 4 weeks) was performed at He Gu, Waiguan, Qu Chi, Da Zhui, Tai Chong, San Yin Jiao, Yang Ling Quan, and Zu San Li acupoints at the National Research Centre (NRC) in Cairo, Egypt. After the treatment, increased plasma superoxide dismutase, glutathione reductase, catalase activities, blood glutathione, and plasma ATP concentrations were observed. Whereas plasma malondialdehyde, serum nitrate and nitrite, serum C-reactive protein, and interleukin-6 levels decreased. A Significant reduction in disease activity was observed based on the DAS28 score [54]. Distal points can be used to treat syndromes that could obstruct Qi, such as Bi syndrome, and needling at local points is applied for adjacent joints [17]. The abovementioned studies elucidated that laser acupuncture, electroacupuncture, and traditional acupuncture were effective in alleviating arthritis-related symptoms.

### 4.6. Stress and Anxiety

Stress can trigger inflammatory responses [55]. It can cause negative outcomes in affected individuals and is usually difficult to treat. A randomized controlled clinical trial with acupuncture decreased the perception of self-identified stress in affected college students, faculty, and staff at the Arizona State University, USA. The acupoints used were Bai Hui, Shen Men, Neiguan, Yintang, Four Gates, Dan Zhong, Qi Hai, and Zu San Li [56]. Heart rate variability increased in 10 patients, aged 33–72 who took acupuncture for hypertension in San Francisco, USA. A decrease in low-frequency–to–high-frequency (LF/HF) ratio of heart rate variability could be correlated to a decrease in physiological stress among the patients. The acupoints used were Zu San Li, Shang Ju Xu, Quchi, Shou San Li, Neiguan, and Da Ling [57]. Elevated inflammation is associated with anxiety [58]. A single acupuncture treatment at bilateral acupuncture point Shen Men was performed in twenty-five male subjects (aged 28 ± 5 years) at the University of Regensburg, Germany. Saliva samples were examined for cortisol and amylase. Anxiety questionnaires and heart rate variability were analyzed. The results indicate that there was a reduction in stress hormones and heart rate compared to existing reference data. Changes in other parameters were not significant [59]. Both auricular acupuncture and placebo interventions reduced the exam anxiety in forty-four medical students of University Medicine of Greifswald, Germany. The acupoints used were MA-IC1 (Lung), MA-TF1 (ear Shenmen), MA-SC (Kidney), MA-AT1 (Subcortex), and MA-TG (Adrenal gland). Yet, auricular acupuncture was effective in reducing exam anxiety as observed by visual analogue scales STAI and VAS-100 [60]. These clinical trials indicate that acupuncture is a possible candidate for the treatment of anxiety and stress.

### 4.7. Crohn's Disease

Moxibustion and acupuncture regulated the ratio of Th17 and Treg cells in the intestinal mucosa of Ninety-two patients with Crohn's disease. Moxibustion was performed on the Tian Shu, Qi Hai, and Zhong Wan acupoints, whereas acupuncture was performed on the Zu San Li, Shang Ju Xu, San Yin Jiao, Tai Xi, Gong Sun, and Tai Chong acupoints [61]. In a case report, a 53-year-old woman suffering from refractory Crohn's disease was reported for treatment with acupuncture. Ququan, Quchi, Zhong Wan, Qi Hai, Zu San Li, and San Yin Jiao were the acupoints used. The frequency of treatment was once per week for a total of 21 sessions. The improvement of symptoms such as chronic indigestion, reflux, abdominal pain, and excessive diarrhea were observed after acupuncture treatment [19]. Ninety-two patients with Crohn's disease were treated with moxibustion at Tian Shu, Qi Hai, and Zhong Wan acupoints. Zu San Li, Shang Ju Xu, Gong Sun, San Yin Jiao, Tai Xi, and Tai Chong were used for acupuncture in various medical centers around Shanghai, China. The hemoglobin increased, whereas the C-reactive protein levels decreased after treatment. The Crohn's disease Activity Index also decreased after treatment [62]. Pure, indirect moxibustion is preferred for clinical treatment of Crohn's disease [61, 63]. These studies determine that acupuncture and moxibustion can have significant therapeutic benefits in patients with Crohn's disease.

The locations of acupoints used for the treatment of inflammation-associated disorders are enlisted in Table 1. The outcomes of acupuncture intervention are enlisted in Table 2.

## 5. Adverse Effects

Acupuncture is considered to be safe and known to possess limited side effects compared to clinical drugs. Although considered safe, the adverse events of acupuncture include the worsening of existing symptoms, dermatitis, fainting, fatigue, bruising, pain, and bleeding at the needling site. Needle breakage may lead to injuries in the lung and spinal cord, local argyria, dizziness, nausea, ocular, and sleeping disorders. Cardiovascular side effects such as endocarditis and tamponade were observed in clinical trials. The pulmonary side effects are pneumothorax and asthma. The intervention could even turn fatal by infections such as hepatitis B [64–68].

## 6. Needling and Duration of Intervention

Needling is performed at specific points to maintain a balance between yin and yang which is ultimate for the circulation of Qi [69]. Needling for 30 minutes is therapeutically effective for acute injuries, whereas 60 minutes is optimum for chronic injuries [70]. The duration of treatment for allergic rhinitis is 4 to 8 weeks [30, 31]. The duration for atherosclerosis was 4 to 10 weeks [47, 48]. For cancer and arthritis, the duration was 2 to 8 weeks and 4 weeks, respectively [50, 53, 54]. The duration of intervention for stress ranged between 2 to 12 weeks [56, 57]. For Crohn's disease, the duration was between 12 to 21 weeks [19, 61, 62]. The predominant duration was 4 weeks for the treatment of chronic inflammation-related diseases using acupuncture as per these reports.

## 7. Challenges and Future Directions

The ambiguous use of terminologies associated with acupuncture points is a critical challenge in therapy. Standard randomized controlled trials that adhere to the STRICTA and CONSORT criteria should be conducted in the future to ensure the effectiveness of acupuncture on chronic inflammation-related disorders. Blinding of parties including the patient and the evaluator are necessary to avoid conscious and unconscious bias in the design and execution of such clinical trials. Needling response should be clearly illustrated along with the experience of the acupuncture practitioner. Rigorous analysis of factors such as the pattern of stimulation, mode and dose, frequency of sessions, and overall duration of the treatment should be performed. Depth of needling is critical. Research on Acupuncture depth still remains underexplored and must be considered mandatory for future research. Mechanistic studies pertaining to peripheral and clinical metrics for determination of the specific effect of acupuncture are the need of the hour. Anti-inflammatory effects of acupuncture are recently analyzed with care and are under the spotlight as acupuncture can possibly modulate inflammation.

## 8. Conclusion

Acupuncture depends on intervention in several acupoints and 12 meridians to maintain the homeostasis of the body through the release of neurotransmitters. Several reports have been published recently on the positive effects of acupuncture with regard to inflammation-oriented disorders. These reports suggest that Neiguan and Zu San Li are common acupoints used for acupuncture intervention of chronic inflammatory diseases. Risk assessment is of paramount importance in the fair, reasonable, and timely use of acupuncture for such diseases. Standard randomized controlled trials that adhere to approved criteria for practice and study the mechanisms involved are therefore the need of the hour to analyze the factors involved and predict the exact mode of action. The current review suggests that acupuncture could be applied to treat inflammation-related disorders in an appropriate manner.

## Figures and Tables

**Table 1:** Location of the acupoints used in the treatment of chronic inflammation-oriented disorders.

S. No.	Acupoint	Abbreviation	Location
1	Bai Hui	GC 20	5 cun posterior to the anterior hairline
2	Chize	LU 5	At the cubital crease on the radial side of the biceps brachii tendon
3	Da Ling	PC 7	In the middle of the wrist crease between the tendons of palmaris ongus and flexor carpi radialis
4	Da Zhui	DU 14	On the midline of the base of the neck, in the depression below the spinous process of the 7th cervical vertebra
5	Dan Zhong	CV 17	Level with the 4th intercostal space, midway between the nipples
6	Fei Shu	BL 13	1.5 cun lateral to the lower border of the spinous process of the third thoracic vertebra (T3)
7	Fengchi	GB 20	In the depression created between the origins of the sternocleidomastoid and trapezius muscles, at the junction of the occipital and nuchal regions
8	Fenglong	ST 40	On the anterior of the leg midway between the crease of the knee and the tip of the lateral malleolus 2 Cun lateral of the tibia
9	Ge Shu	BL 17	1.5 cun lateral to the lower border of the spinous process of the seventh thoracic vertebra (T7)
10	Gong Sun	SP 4	On the medial aspect of the foot, in the depression distal and inferior to the base of the first metatarsal bone
11	Guan Yuan	CV 4	On the anterior midline of the lower abdomen, 3 cun below the umbilicus
12	He Gu	LI 4	On the dorsum of the hand, between the 1st and 2nd metacarpal bones, in the middle of the 2nd metacarpal bone on the radial side
13	Hou Xi	SI 3	When a loose fist is made, the point is on the ulnar aspect of the hand, proximal to the 5th metacarpophalangeal joint, at the end of the transverse crease of the metacarpophalangeal joint, at the junction of the red and white skin
14	Jing Men	GB 25	On the lateral side of the abdomen on the lower border of the free end of the 12th rib
15	Lie Que	LU 7	On the radial margin of the forearm, superior to the styloid process of the radius, 1.5 cun above the transverse crease of the wrist
16	Neiguan	PC 6	Three finger breadths below the wrist on the inner forearm in between the two tendons
17	Pishu	BL 20	1.5 cun lateral to the posterior midline, on the level of the lower border of the spinous process of the 11th thoracic vertebra (T11)
18	Qi Hai	CV 6	On the midline, 1.5 cun inferior to the umbilicus
19	Quchi	LI 11	When the elbow is flexed, the point is in the midpoint between the lateral end of the transverse cubical crease and the lateral epicondyle of the humerus
20	Ququan	LV 8	On the medial aspect of the knee, when the knee is flexed, the point is in the depression on the medial end of the transverse popliteal crease, on the posterior border of the medial epicondyle of the femur, on the anterior portion of the insertion of semitendinosus and semimembranosus muscles
21	Renying	ST 9	Level with the tip of the Adam's apple on the anterior border of the sternocleidomastoideus muscle (where the pulse of the common carotid artery is felt)
22	San Yin Jiao	SP 6	On the inside of your leg, just above your ankle
23	Shang Ju Xu	ST 37	3 cun below Zu San Li, one finger breadth from the anterior crest of the tibia, in muscle tibialis anterior
24	Shang Xing	GV 23	At the top of the head, 1 cun posterior to the midline of the anterior hairline
25	Shang Yang	LI 1	.1 cun posterior to the corner of the nail on the radial side of the index finger
26	Shao Shang	LU 11	.1 cun posterior to the thumb nail on the radial side
27	Shen Men	HT 7	At the wrist crease, on the radial side of the flexor carpi ulnaris tendon, between the ulna and the pisiform bones
28	Shen Shu	BL 23	1.5 cun lateral to the posterior midline, on the level of the lower border of the spinous process of the 2nd lumbar vertebra (L2)
29	Shou San Li	LI 10	On the outer surface of the forearm and three fingers breadth below the elbow crease when the elbow is bent 90 degrees
30	Sibai	ST 2	With the eyes looking straight ahead, directly below the centre of the pupil, in the depression at the infraorbital foramen
31	Tai Chong	LV 3	On the dorsum of the foot, in a depression distal to the junctions of the first and second metatarsal bones
32	Tai Xi	KI 3	On the medial aspect of the foot, posterior to the medial malleolus, in the depression between the tip of the medial malleolus and tendo calcaneus
33	Tai Yuan	LU 9	At the wrist crease on the radial side of the radial artery
34	Tian Shu	ST 25	On the middle of the abdomen, 2 cun lateral to the umbilicus
35	Waiguan	TE 5	2 cun proximal to the dorsal wrist crease between the radius and ulna, close to the radial bone
36	Wan Gu	SI 4	On the ulnar aspect of the palm, in the depression between the 5th metacarpal bone and hamate bone, at the junction of the red and white skin
37	Xiaguan	ST 7	On the face, anterior to the ear, in a depression between the zygomatic arch and the mandibular notch, with mouth closed
38	Ximen	PC 4	5 cun above the wrist crease between the tendons of palmaris longus and flexor carpi radialis
39	Xuan Zhong	GB 39	On the lateral aspect of the lower leg, 3 cun above the tip of the external malleolus, on the anterior border of the fibula
40	Xue Hai	SP 10	When the knee is flexed, on the medial aspect of the thigh, the point is 2 cun above the mediosuperior border of the patella, on the bulge of the medial portion of m. quadriceps femoris
41	Yang Ling Quan	GB 34	In the depression anterior and inferior to the small head of the fibula
42	Ying Ling Quan	SP 9	On the lower border of the medial condyle of the tibia in the depression posterior and inferior to the medial condyle of the tibia
43	Ying Xiang	LI 20	In the nasolabial groove, level with the midpoint of the lateral border of the ala nasi
44	Yintang	EX-HN3	At the forehead, at the midpoint between the two medial ends of the eyebrow
45	Yuji	LU 10	Midpoint of the palmar border of the 1st metacarpal bone
46	Yunmen	LU 2	On the latero-superior aspect of the chest, superior to the coracoid process of scapula, in the depression of the infraclavicular fossa, 6 cun lateral to the anterior median line
47	Zhaohai	KD 6	In a depression below the tip of the medial malleolus
48	Zhong Wan	CV 12	On the midline, 4 cun superior to the umbilicus
49	Zhongfu	LU 1	6 cun lateral to the anterior midline, level with the 1st intercostal space
50	Zu san Li	ST 36	Below the knee, on the tibialis anterior muscle, along the stomach meridian

Note: one cun is equal to the space between the distal interphalangeal joint and the proximal interphalangeal joint on the middle finger. Information of acupoint locations were obtained from the following websites: https://tcmwiki.com/,https://tcmwiki.com/, https://theory.yinyanghouse.com/acupuncturepoints/, https://exploreim.ucla.edu/, https://www.sacredlotus.com/go/acupuncture/, https://www.acatcm.com/, https://www.acufinder.com/Acupuncture+Points/, http://acupunctureschoolonline.com/, Retrieved on 24.01.2020.

**Table 2:** Outcomes of acupuncture treatment in clinical trials for inflammation-associated disorders.

Method	Meridians	Outcome	Country	Ref
Allergic rhinitis				
AC	Xiaguan	Improvement of nasal obstruction and sneezing	China	11
AC	Ying Xiang, Yintang, and Fengchi	Improvement of nasal and nonnasal symptoms	Australia	12
AC	Ying Xiang, Shang Xing, Fengchi, Quchi, Xue Hai, Fei Shu, Ge Shu, and Pishu	Effects of acupuncture treatment were similar to that of oral cetirizine hydrochloride treatment	China	13
AC	He Gu, Ying Xiang, Sibai, Zu San Li, Yintang, and Shang Xing	Reduction in total nasal and nonnasal symptom score	South Korea and China	14
Asthma				
AC	Yuji	Acupuncture effects comparable to the effects of 200 *μ*g salbutamol aerosol	China	18
AC	Zhongfu, Chize, Tai Yuan, Guan Yuan, Zhong Wan, Fei Shu, Pishu, and Shen Shu	Improved the symptoms of asthma and VAS of dyspnea	Japan	20
Atherosclerosis				
EA	Neiguan, Lie Que, and Yunmen	Limited troponin I release, reduction in ICU stay time, and decreased use of inotropic agents	China	21
AC and Moxi	Renying, Neiguan, and Zu San Li	Increase in size of common carotid artery, reduction of intima-media thickness, accelerated peak systolic and end diastolic velocities, decreased pulsatility index, and resistance index	China	22
EA	Neiguan and Ximen	Decrease of the rate of major adverse cardiac/cerebrovascular events after a two-year follow-up	China	23
Cancer				
AC with herbs of TCM	Tai Chong, Zu San Li, He Gu, Qi Hai, Fenglong, and San Yin Jiao	Regression of ductal carcinoma over a period of 15 months	USA	26
AC	Zu San Li, Neiguan, Ge Shu, Fei Shu, Guan Yuan, and Shen Shu	Significant increase of IL-2, CD3+ and CD4+ T cells, and NK cells were observed	China	27
AC	Zu San Li, Quchi, and Qi Hai	Increase in IL-2 level and NK cell activities	China	29
Rheumatoid arthritis				
EA and AC	Quchi, Waiguan, He Gu, Zu San Li, Yang Ling Quan, and Xuan Zhong	Reduction of pain score	China	30
LA	Tai Chong, Tianshu, Zu San Li, Houxi, Wan Gu, He Gu, Qu Chi, San Yin Jiao, Ying Ling Quan, Jing Men, Yang Ling Quan, and Shen Men	Interleukin-6 and malondialdehyde levels decreased, whereas ATP levels and pain-free range of motion at joints increased	Egypt	31
LA	He Gu, Waiguan, Qu Chi, Da Zhui, Tai Chong, San Yin Jiao, Yang Ling Quan, and Zu San Li	Increase in plasma superoxide dismutase, glutathione reductase, catalase activities, blood glutathione and plasma ATP concentrations. Plasma MDA, serum nitrate and nitrite, serum CRP, interleukin-6 levels decreased. Significant reduction in disease activity was observed based on DAS28 score.	Egypt	32
Stress and anxiety				
AC	Bai Hui, Shen Men, Neiguan, Yintang, Four Gates, Dan Zhong, Qi Hai, and Zu San Li	Decrease in the perception of self-identified stress	USA	34
AC	Zu San Li, Shang Ju Xu, Quchi, Shou San Li, Neiguan, and Da Ling	Decrease in low-frequency–to–high-frequency (LF/HF) ratio of heart rate variability	USA	35
AC	Shen Men	Reduction in stress hormones and heart rate	Germany	37
AC	MA-IC1 (lung), MA-TF1 (ear Shenmen), MA-SC (kidney), MA-AT1 (subcortex), and MA-TG (adrenal gland)	*Reduced exam anxiety*	Germany	38
Crohn's disease				
Moxi and AC	Tian Shu, Qihai, Zhongwan, Zu San Li, Shang Ju Xu, San Yin Jiao, Tai Xi, Gong Sun, and Tai Chong	Decrease in the ratio of Th17 and Treg cells	China	39
AC	Ququan, Quchi, Zhong Wan, Qi Hai, Zu San Li, and San Yin Jiao	Improvement of symptoms such as chronic indigestion, reflux, abdominal pain, and excessive diarrhea	China	40
Moxi and AC	Tian Shu, Qi Hai, Zhong Wan, Zu San Li, Shang Ju Xu, Gong Sun, San Yin Jiao, Tai Xi, and Tai Chong	Increased hemoglobin levels. Decreased CRP levels and Crohn's disease activity index	China	41

AC: acupuncture; EA: electroacupuncture; Moxi: moxibustion; LA: laser acupuncture.
